# Is there a shift from cardiovascular to cancer death in lipid-lowering trials? A systematic review and meta-analysis

**DOI:** 10.1371/journal.pone.0297852

**Published:** 2024-02-08

**Authors:** Lucy Bolt, Alexandre Speierer, Sylvain Bétrisey, Martina Aeschbacher-Germann, Manuel R. Blum, Baris Gencer, Cinzia Del Giovane, Drahomir Aujseky, Elisavet Moutzouri, Nicolas Rodondi

**Affiliations:** 1 Institute of Primary Health Care (BIHAM), University of Bern, Bern, Switzerland; 2 Department of General Internal Medicine, Inselspital, Bern University Hospital, University of Bern, Bern, Switzerland; 3 Department of Cardiology, Geneva University Hospital (HUG), University of Geneva, Geneva, Switzerland; HT Ong Heart Clinic, MALAYSIA

## Abstract

**Background:**

Lipid-lowering therapy (LLT) reduces cardiovascular (CV) events, but data are conflicting on all-cause mortality, especially among older adults. Though LLT does not induce cancer, some randomized clinical trials (RCTs) found a pattern of increased cancer death under LLT. Our objective was to assess a possible shift from CV to cancer death in LLT trials (i.e. an increase in cancer and decrease in CV death) and to investigate potential subgroups at risk.

**Methods:**

We performed a systematic review and meta-analysis. We retrieved RCTs from MEDLINE, Embase, and Cochrane Central until 08/2023. We extracted the number of CV and cancer deaths in the treatment vs. in the control arm, calculated the relative risk (RR) by dividing the risk of death in the treatment over the risk of death in the control group and then pooled them using random-effect meta-analysis. We performed subgroup analyses on primary and secondary prevention, and according to different age cut-offs.

**Results:**

We included 27 trials with 188’259 participants (23 statin; 4 ezetimibe trials). The trials reported 4056 cancer deaths, 2061 under LLT and 1995 in control groups. Overall, there was no increased risk of cancer mortality (RR 1.03, 95% confidence interval 0.97–1.10), with no difference between primary and secondary prevention. In the subgroup analyses for RCTs with ≥15% of participants aged ≥75 years, the RR of cancer death was 1.11 (1.00–1.23), while the RR for CV death was 0.96 (0.91–1.01). For RCTs with a mean age ≥ 70 years, the RR for cancer death was 1.21 (0.99–1.47).

**Conclusion:**

LLT does not lead to a shift from CV to cancer death. However, there might be a possible shift with a pattern of increased cancer deaths in trials with more older adults, particularly ≥75 years. Individual participant data from LLT trials should be made public to allow further investigations.

**PROSPERO registration:**

CRD42021271658

## Introduction

Lipid-lowering therapy (LLT) has been shown to reduce major vascular events in a wide range of patient groups, but data are conflicting for all-cause mortality, especially among older people in primary prevention [1]. Three previous randomized clinical trials (RCTs) have found a pattern of increased cancer death in the group treated with LLT, although without statistical significance [2–4]. For example, in the main ALLHAT-LLT trial on pravastatin vs. usual care, there were 163 cancer related deaths in pravastatin vs. 148 in usual care (relative risk (RR) 1.11 (95% confidence interval (CI), 0.89–1.39)) in the overall population [4]; in a secondary analysis of ALLHAT-LLT participants in primary prevention, the hazard ratio (HR) for all-cause mortality in the pravastatin group vs. the control group were 1.18 (95% CI, 0.97–1.42) for ≥65 years, 1.08 (95% CI, 0.85–1.37) for 65–74 years, and 1.34 (95% CI, 0.98–1.84) for adults ≥75 years, while specific cancer deaths for primary prevention were not reported (cancer, fatal and nonfatal: 131 in pravastatin vs. 113 in usual care (HR 1.14, 95% CI, 0.88–1.46) [5]. The authors noted “*No benefit was found when pravastatin was given for primary prevention to older adults with moderate hyperlipidemia and hypertension*, *and a non-significant direction toward increased all-cause mortality with pravastatin was observed among adults 75 years and older*” [5]. As meta-analyses have found that statins do not induce cancer or increase cancer death [1], the question remains on the potential reasons for this pattern of a non-significant increased all-cause mortality and cancer death observed in some RCTs with LLT. A possible explanation is that LLT treatment induces a shift from cardiovascular (CV) death to cancer death, so that a decrease in CV mortality may be counterbalanced by an increase in cancer mortality [6]. We based our hypothesis on above mentioned data and the context of US epidemiological data showing a transition from CV disease to cancer as the leading cause of death in the USA [6].

The purpose of this systematic review and meta-analysis was to assess a possible shift from CV to cancer death in LLT trials in primary and secondary prevention and to investigate potential subgroups at risk. Our main hypothesis was that older adults taking statins would die less of CV causes and more of cancer, compared to non-takers.

## Materials and methods

We followed the Preferred Reporting Items for Systematic reviews and Meta-Analyses (PRISMA) statement [7] (S1 Checklist) and published the protocol (S1 File) of this systematic review on PROSPERO (CRD42021271658).

### Eligibility criteria and literature search

We considered RCTs with LLT reporting any CV clinical events as primary endpoints in primary and secondary prevention. We excluded pseudo randomization (e.g. pre-post comparisons). The intervention had to consist of LLT defined as statin, ezetimibe, and proprotein convertase subtilisin/kexin type 9 (PCSK 9) inhibitor (or a combination), as these drugs have been shown to reduce CV risk [8], with a minimum follow-up of two years. The control group had to receive either placebo or no therapy. To be included, RCTs had to include a minimum of 1000 participants and to report quantitative data of both CV death and cancer death. The cut-off of 1000 participants was chosen in accordance with the Cholesterol Treatment Trialists’ Collaboration (CTTC) [9]. Furthermore, we aimed to avoid some possible small study effects biases and to include homogenous major cardiovascular trials. As death from cancer is fortunately not a very common outcome in trials conducted over a few years, we aimed to avoid some random differences, which could be attributed to differences in trial size. Data had to be reported with effect estimates and measures of precision (standard deviations or standard errors). We excluded duplicate data, secondary subgroup trial data analyses, post-trial follow up studies and studies with low-density cholesterol (LDL-C) as primary endpoint.

We first retrieved all trials from the CTTC [9]. As the last trial included in the CTTC analysis was published in 2016, we further systematically searched MEDLINE, Embase, and Cochrane Central between January 1, 2015 ‐ May 25, 2021 and completed an updated search on August 16, 2023 in cooperation with a trained librarian, as previously described [10]. Search terms were adapted according to the syntax of each specific database and no language restrictions were applied. Details of our search strategy can be found as supplementary material (S1 Data).

### Study selection

In a first step, three authors (LB, AS and MA) screened the study titles and abstracts for eligibility. In a second step, the same authors screened the full-text of studies eligible from step one. The two first steps were done independently. Disagreement was discussed and, when no consensus was found, an additional independent person was consulted (EM). The study selection process was conducted using the software Rayyan [11]. Excluded studies evaluated in full-text were listed, with the reasons for exclusion being specified. Discrepancies were resolved by consensus among the study team.

### Data extraction and risk of bias assessment

A standard data extraction form was used, adapted from a template suggested by Cochrane [12]. We extracted bibliographic details; eligibility criteria; information on the study population and setting; study design; intervention/control intervention; outcome data (number of participants in each group, follow-up time, the number of CV death, cancer death and overall death); and data on statistical analysis performed. One second author (MA, SB, LB, EM) independently reviewed the extraction. For risk of bias assessment, we used RoB 2.0 according to Cochrane Collaboration 2019 [13] which was performed by two authors independently (AS and MA) and for the updated search by AS and LB (S1 Fig). A third author (EM) was involved in case of discrepancies.

### Statistical analyses

To assess a possible shift in death causes, study results were presented separately for each outcome (CV death and cancer death). We used the RR as relative treatment effect with relative 95% CIs. We did not use HRs as we were not interested in time to death (which was also not provided by most trials, particularly for cancer death), but in a possible shift in the causes of death. We calculated the risk of cancer death in each group by dividing the absolute number of cancer deaths (extracted from each trial) with the overall population in the respective group. We calculated the RR of cancer death by dividing the risk of cancer death in the treatment group by the risk of cancer death in the control group. We used the same approach for the RR of CV death. A RR above 1 would mean an increased risk of death in the treatment group compared with the control. We combined the results across studies for each outcome by using a random effect model. Heterogeneity was assessed visually with forest plots and quantified with I^2^.

### Subgroup & sensitivity analyses

Subgroup analyses were performed on primary vs. secondary prevention, on trials with different prevalence of participants aged ≥75 years (10%, 15%, 20% and 25%) given the current controversial discussion on statins among adults aged ≥75 years [8, 14], and on trials with a mean age over or under 70 years. We did sensitivity analyses excluding trials with high risk of bias and with premature end. We also did sensitivity analyses including trials with statins only. P-values were two-sided and considered significant at p<0.05. All analyses were conducted in Stata version 16.0 (Stata Corporation, College Station, Texas).

### Ethics approval statement

An ethics approval statement was not needed for the purpose of this study.

## Results

**Fig 1:**
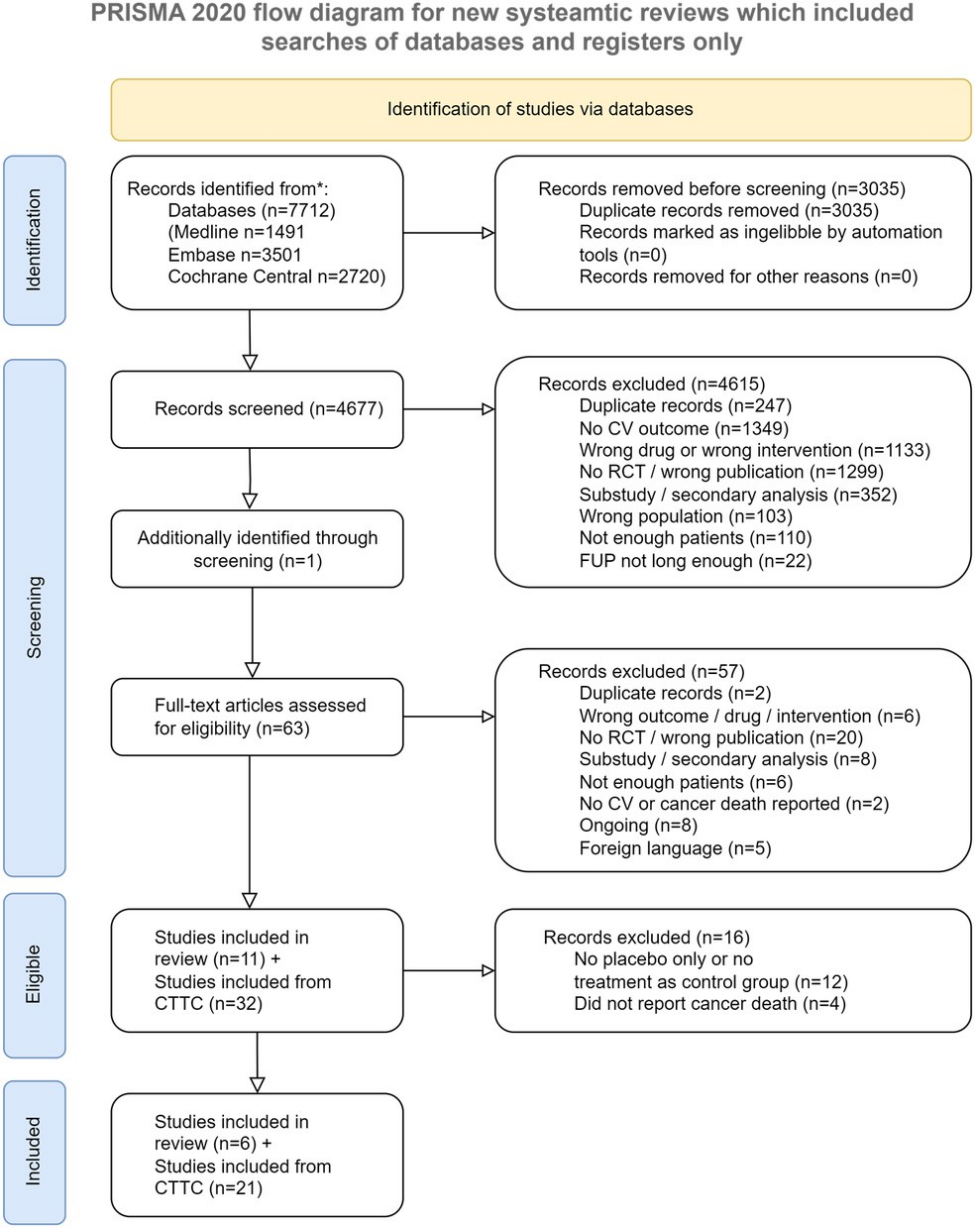
Flow chart of included trials. CV = cardiovascular, RCT = randomized controlled trial, FUP = follow-up period, CTTC = Cholesterol Treatment Trialists’ Collaboration.

We identified 43 trials, of which 12 trials did not have a placebo arm only or a no-treatment arm as control group. PCSK 9-inhibitor trials [15, 16] and the only large bempedoic acid trial [17] did not report cancer death. We therefore included 27 trials (21 trials from the CTTC and 6 additional trials/23 statin trials and 4 ezetimibe trials) (Fig 1 and S1 Table) with a total of 188’259 patients. In total, 23 trials used placebo, one trial used no treatment, and three trials used usual care or diet modification as their control arm.

**Fig 2:**
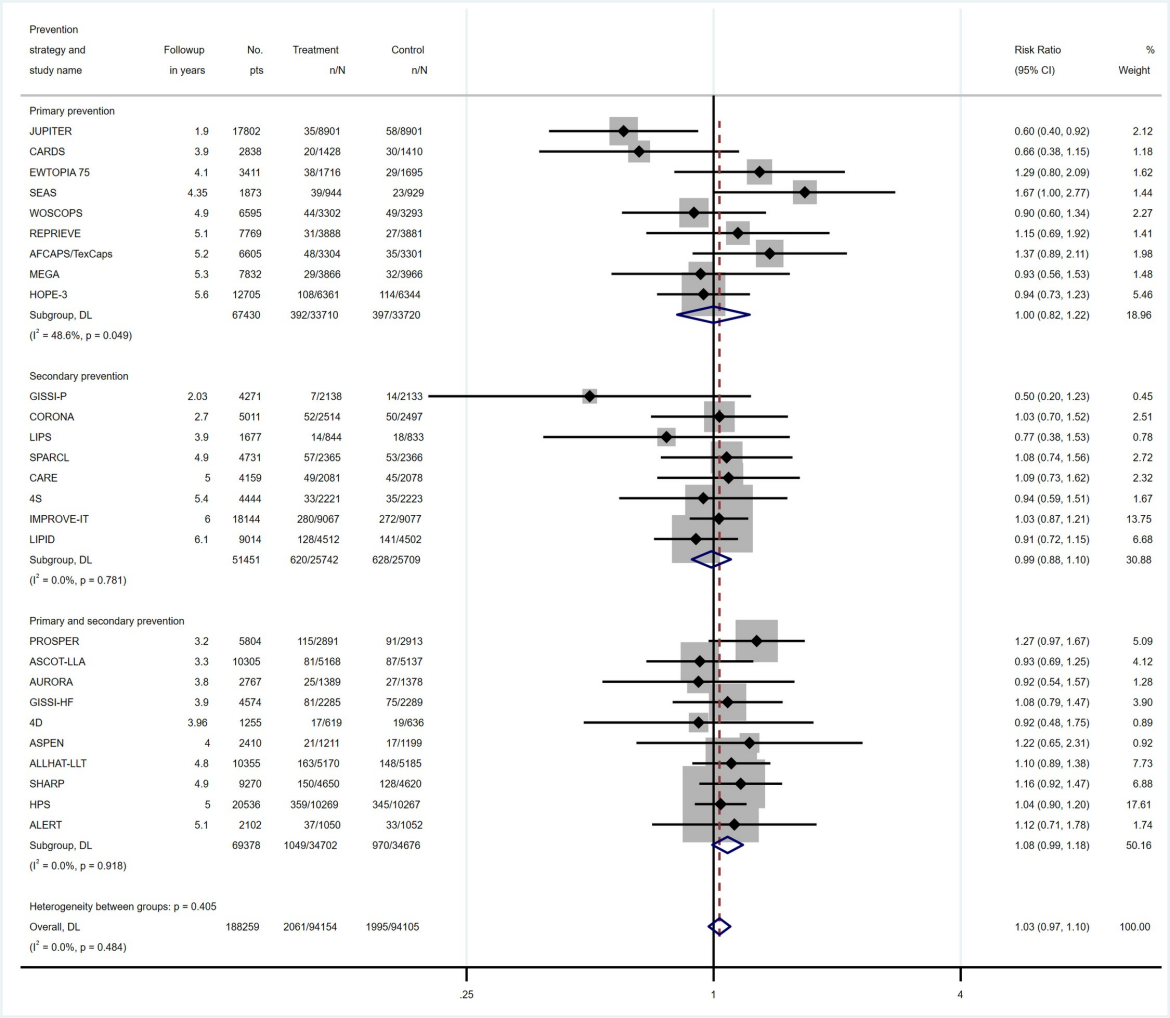
Relative risk for cancer mortality according to lipid-lowering therapy and prevention strategy. pts = participants, CI = confidence interval.

Overall, the 27 included trials reported 4056 cancer deaths, 2061 in the treatment arm and 1995 in the control groups (S1 Table). Overall, there was no statistically significant increase in cancer mortality (RR 1.03, 95% CI, 0.97–1.10). The RR for cancer mortality was 1.00 (95% CI, 0.82–1.22) in primary, 0.99 (95% CI, 0.88–1.10) in secondary, and 1.08 (95% CI 0.99–1.18) in RCTs assessing both primary and secondary prevention (Fig 2). In total, 10434 CV deaths were reported (4918 in the treatment arm, 5516 in the control arm) (S1 Table). The RR for CV death was significantly reduced in the three groups (RR 0.78, 95% CI, 0.69–0.90; RR 0.83, 95% CI, 0.74–0.94 and RR 0.92, 95% CI, 0.88–0.97, respectively, S2 Fig). In sensitivity analyses, results for cancer mortality did not change by excluding high risk of bias trials or trials with premature end and including only statin trials (S2 Table). Only one trial adjusted their results using a competing risk model (EWTOPIA 75) [2].

**Fig 3:**
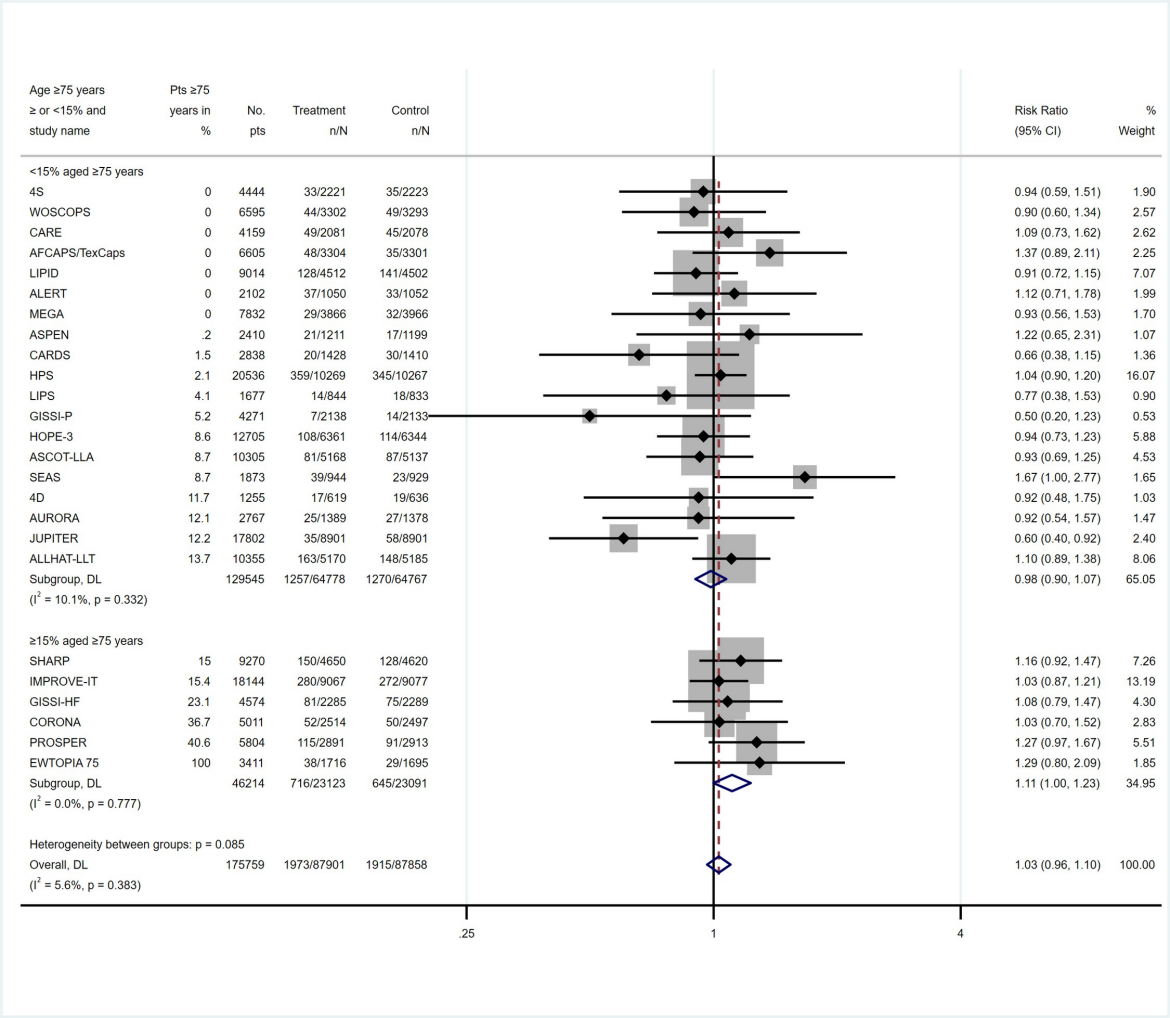
Relative risk for cancer mortality according to lipid-lowering therapy and the prevalence of participants aged ≥75 years^1 1^. The SPARCL and REPRIEVE trial did not report the prevalence of participants aged ≥75 years pts = participants, CI = confidence interval.

In the subgroup analysis of trials with more than 15% of the participants aged ≥75 years, the RR of cancer death was 1.11 (95% CI, 1.00–1.23) (Fig 3), while the RR for CV death was 0.96 (95% CI, 0.91–1.01) (S3 Fig). The RR for cancer mortality for other prevalence of participants aged ≥75 years are reported in Table 1. As SPARCL and REPRIEVE did not report the prevalence of adults ≥75 years, they could not be included in this subgroup analysis. SPARCL reported a HR of 1.05 (95% CI, 0.72–1.53) for cancer death [18]. In the analysis of trials with a mean age ≥70 years compared to those with a mean age <70 years, the RR for cancer death was 1.21 (95% CI, 0.99–1.47) (S4 Fig).

**Table 1:** Relative risk for cancer and cardiovascular death according to different cut-offs for prevalence of patients aged ≥75 years^1^.

	Cancer death		CV death	
Cut-off	< cut-off	≥ cut-off	< cut-off	≥ cut-off
	RR (95% CI)			
10%	1.00 (0.92–1.09)	1.07 (0.96–1.18)	0.80 (0.76–0.85)	0.96 (0.92–1.01)
15%	0.98 (0.90–1.07)	1.11 (1.00–1.23)	0.85 (0.80–0.90)	0.96 (0.91–1.01)
20%	1.01 (0.94–1.08)	1.17 (0.99–1.38)^b^	0.87 (0.82–0.92)	0.95 (0.86–1.04)

CV = cardiovascular, RR = risk ratio, CI = confidence interval

^1^The SPARCL and REPRIEVE trial could not be included in this analysis because of missing data on the prevalence of older adults ≥75 years

^b^ P for trend 0.06

To address the issue of cancer latency and to avoid some unreported imbalances at cancer at baseline between groups of interest, we performed a sensitivity analysis including only trials, which excluded patients with a cancer diagnosis at baseline. In total, n = 20 trials excluded people with cancer at baseline and were included in these sensitivity analyses. These analyses did not show any significant changes in the results, compared to our main analyses (S5–S7 Figs).

## Discussion

In this systematic review of 27 large RCTs on LLT, we found no clear evidence for a shift from CV death to cancer death in LLT takers, compared to non-takers. However, our subgroup analysis on trials with a higher prevalence of participants aged ≥75 years showed a possible shift with a pattern of increased risk of cancer death. A further subgroup analysis including trials with participants with a mean age of ≥70 years showed a similar pattern.

Causes of death represent mutually exclusive outcomes in RCTs and the different associations with specific treatments could be particularly of interest, especially in older adults, as rates of mortality are much higher than in younger groups. Some previous individual RCTs have shown a pattern of increased cancer death and all-cause mortality in participants treated with LLT [2–4]. The individual participant meta-analysis performed by the CTTC showed no significant difference for cancer death with a risk rate per 1mmol/L reduction in LDL-C of 0.99 (95% CI, 0.93–1.06) for the overall population (similar to our results), 1.09 (95% CI 0.92–1.29) among older adults aged 70–75 years and 0.92 (95% CI 0.71–1.18) ≥75 years [1]. However, we further included trials conducted after 2015 that were not in the CTTC report [2] and the effect measure used was HR calculated per 1mmol/L reduction in LDL-C applied for time to event outcome, while we were not interested in time to death, but a possible shift in the causes of death, with the hypothesis that patients under statins dying less from CV causes, might die more from cancer deaths, as this pattern was found in 3 previous RCTs [2–4]. In addition, we included only trials that did not use LLT in the control arm and included six additional trials [2, 19–23] compared to the CTTC report [9], which allows a better assessment of benefits and harms of LLT, as well as studies including ezetimibe.

Among limitations, specific numbers of events on cancer death were, unfortunately, not available at study level for the subgroups of the older adults (≥70 years or ≥75 years), which forced us to assess trials according to the proportion of older adults (≥70 years or ≥75 years). An important limitation of our systematic review is the use of aggregated data with a potential risk of ecological fallacy, although three individual trials also reported such a potential shift with a pattern of increased cancer death under LLT [2–4]. In addition, most RCTs did not provide mean follow-up time separately for treatment and control groups to allow performing a competing risk model using aggregated data, to be able to assess the proportion of cause-specific death overtime [24]. In our protocol, we planned to estimate the cumulative incidence functions RR to measure the treatment effect over time, taking into account the probability of each outcome. However, for this analysis we would have needed the total patient-time per treatment arm per study in each RCT until cancer death, which was not reported. Another limitation of our study are the age cut-offs for the subgroup analyses. As we did not know the type of data available in each study before the analyses, we predefined our subgroup analyses to the older population without specifying the age cut-offs. Given the current controversial discussion on statins and older adults, defined as ≥75 years, with most uncertainty for statin use [8, 14], it led us to choose this cut-off. Results were also similar in the analysis of trials with mean age ≥70 years to ensure that at least half the participants were older than 70 years. To allow further analyses, individual participant data from LLT trials should be made publicly available, which is currently not the case [25, 26]. Also, we find unfortunate the fact that recent PCSK 9-inhibitor trials and the one large bempedoic acid trial have not published cancer deaths [15–17].

## Conclusion

The present meta-analysis of aggregated mortality data shows that lipid-lowering drugs reduce CV death, which are 2.5 more common than cancer death in these patients. However, the point estimates yielded a larger increase in cancer deaths in trials with more older adults, with a possible shift with a pattern of increased cancer deaths in trials with more older adults, particularly ≥75 years. These findings are hypothesis generating and should be further investigated with individual participant data from LLT trials that should be made publicly available [25, 27]. Further research on statins should include more older participants, particularly ≥70 and 75 years, to further clarify this controversy, as done by ongoing trials [28, 29].

## Supporting information

S1 Checklist (DOC)

S1 Data Search strategy. (DOCX)

S1 Dataset (XLS)

S1 File Prospero protocol. (DOCX)

S1 Fig Risk of bias for the included trials (Rob 2.0) ^1^. ^1^ Green represents a low, orange an intermediate, and red a high risk of bias.
(TIF)

S2 Fig Relative risk for cardiovascular mortality according to primary and secondary prevention. ^1^ pts = participants, CI = confidence interval, ^1^ Weights and between-subgroup heterogeneity test are from random-effects model.
(TIF)

S3 Fig Relative risk for cardiovascular mortality according to prevalence of older participants (≥75 years) in primary and secondary prevention. ^1, 2^ pts = participants, CI = confidence interval, ^1^Weights and between-subgroup heterogeneity test are from random-effects model, ^2^The SPARCL and REPRIEVE trial did not report the prevalence of participants aged ≥75 years.
(TIF)

S4 Fig Relative risk for cancer mortality according to prevalence of older participants (mean age ≥70 years) in primary and secondary prevention^1, 2^. pts = participants, CI = confidence interval, ^1^ Weights and between-subgroup heterogeneity test are from random-effects model, ^2^The HPS trial did not report the mean age.
(TIF)

S5 Fig Relative risk for cancer mortality according to lipid-lowering therapy and prevention strategy in trials excluding cancer at baseline^1^. pts = participants, CI = confidence interval, ^1^ Weights and between-subgroup heterogeneity test are from random-effects model.
(TIF)

S6 Fig Relative risk for cancer mortality according to lipid-lowering therapy and the prevalence of participants aged ≥75 years in trials excluding cancer at baseline^1^. pts = participants, CI = confidence interval, ^1^The REPRIEVE trial did not report the prevalence of participants aged ≥75 years.
(TIF)

S7 Fig Relative risk for cancer mortality according to prevalence of older participants (mean age ≥70 years) in primary and secondary prevention in trials excluding cancer at baseline^1, 2^. pts = participants, CI = confidence interval, ^1^Weights and between-subgroup heterogeneity test are from random-effects model, ^2^The HPS trial did not report the mean age.
(TIF)

S1 Table Baseline characteristics. LLT = Lipid-lowering treatment, CV = cardiovascular.
(DOCX)

S2 Table Sensitivity analyses of relative risk for cancer mortality for different trial criteria by prevention strategies. RR = relative risk, CI = confidence interval.
(DOCX)
