# Supplementary material for: Is there a shift from cardiovascular to cancer death in lipid-lowering trials? A systematic review and meta-analysis
Source: PLoS One. 2024 Feb 8;19(2):e0297852. doi: 10.1371/journal.pone.0297852 (PMC10852259; doi:10.1371/journal.pone.0297852)
Supplement: S1 Data — (DOCX) [file pone.0297852.s002.docx]

### **S1 Data.**

Date of last search **25th May 2021/ updated 16th August 2023**

**Ovid MEDLINE**(R) and Epub Ahead of Print, In-Process, In-Data-Review & Other Non-Indexed Citations, Daily and Versions(R) <1946 to May 24, 2021> & for update: <2021 to August 16, 2023>

1 exp cholesterol/

2 (cholesterol* or lipid* or LDL).ab,ti,kf.

3 1 or 2

4 exp Anticholesteremic Agents/

5 ((inhibit* adj3 ("hmg-coa*" or "Hydroxymethylglutaryl CoA*" or "Hydroxymethylglutaryl-Coenzyme A")) or statins or statin or simvastatin or rosuvastatin or pravastatin or pitavastatin or mevastatin or lovastatin or glenvastatin or fluvastatin or fluindostatin or dalvastatin or crilvastatin or atorvastatin or cerivastatin or bervastatin or medostatin).ab,kf,ti,nm.

6 (altoprev or altocor or baycol or canef or cranoc or compactin or crestor or lescol or lipitor or lipex or lipostat or livalo or locol or lochol or mevinolin or mevacor or mevalotin or mevinacor or monacolin or pravachol or pitava or pravachol or pravasin or zocor).mp.

7 (antichol* or antihyperchol* or hypochol* or hypolipidemic* or antihyperlipidemic* or anti-hyperlipidemic* or Ezetimibe or PCSK9 inhibitor* or Alirocumab or evolocumab or non-statin*).ab,ti,kf.

8 4 or 5 or 6 or 7

9 exp cardiovascular diseases/ or exp mortality/

10 (((cardiovascular or heart or coronar* or cardiac) adj3 (disease* or event* or attack* or mortalit* or death* or arrest*)) or cvd or cvds or CV-mortalit* or MACE or angina or ((heart or cardia* or myocard*) adj3 (ischemi* or ischaemi* or fail* or insufficien*)) or ((myocard* or heart or cardiac) adj3 (infarct* or attack*)) or (cerebrovascular* adj3 (accident* or event*)) or cva or stroke* or ((brain or cerebral) adj3 (ischemi* or ischaemi*))).ti,ab.

11 9 or 10

12 3 and 8 and 11

13 randomized controlled trial.pt.

14 (random$ or placebo$ or single blind$ or double blind$ or triple blind$).ti,ab.

15 (retraction of publication or retracted publication).pt.

16 or/13-15

17 (animals not humans).sh.

18 ((comment or editorial or meta-analysis or practice-guideline or review or letter) not randomized controlled trial).pt.

19 (random sampl$ or random digit$ or random effect$ or random survey or random regression).ti,ab. not randomized controlled trial.pt.

20 16 not (17 or 18 or 19)

21 12 and 20

22 limit 21 to yr="2015 -Current" / for update: limit 21 to yr="2021 -Current"

mp=(multi-purpose field): title, abstract, original title, name of substance word, subject heading word, floating sub-heading word, author keyword heading word, organism supplementary concept word, protocol supplementary concept word, rare disease supplementary concept word, unique identifier, synonyms

nm=Name of substance word
kf=Author keyword heading word

BMJ Best Practice Study design search filters
Medline randomised controlled trial strategy
<https://bestpractice.bmj.com/info/toolkit/learn-ebm/study-design-search-filters/>

**Embase** **(Ovid)** <1974 to 2021 May 24> & for update: <2021 to August 16, 2023>

1 cholesterol/

2 (cholesterol* or lipid* or LDL).ti,ab,kw.

3 1 or 2

4 exp hypocholesterolemic agent/

5 ((inhibit* adj3 ("hmg-coa*" or "Hydroxymethylglutaryl CoA*" or "Hydroxymethylglutaryl-Coenzyme A")) or statins or statin or simvastatin or rosuvastatin or pravastatin or pitavastatin or mevastatin or lovastatin or glenvastatin or fluvastatin or fluindostatin or dalvastatin or crilvastatin or atorvastatin or cerivastatin or bervastatin or medostatin).ab,kw,ti,rn.

6 (altoprev or altocor or baycol or canef or cranoc or compactin or crestor or lescol or lipitor or lipex or lipostat or livalo or locol or lochol or mevinolin or mevacor or mevalotin or mevinacor or monacolin or pravachol or pitava or pravachol or pravasin or zocor).mp.

7 (antichol* or antihyperchol* or hypochol* or hypolipidemic* or antihyperlipidemic* or anti-hyperlipidemic* or Ezetimibe or PCSK9 inhibitor* or Alirocumab or evolocumab or non-statin*).ti,ab,kw.

8 4 or 5 or 6 or 7

9 exp cardiovascular disease/ or exp mortality/

10 (((cardiovascular or heart or coronar* or cardiac) adj3 (disease* or event* or attack* or mortalit* or death* or arrest*)) or cvd or cvds or CV-mortalit* or MACE or angina or ((heart or cardia* or myocard*) adj3 (ischemi* or ischaemi* or fail* or insufficien*)) or ((myocard* or heart or cardiac) adj3 (infarct* or attack*)) or (cerebrovascular* adj3 (accident* or event*)) or cva or stroke* or ((brain or cerebral) adj3 (ischemi* or ischaemi*))).ti,ab.

11 9 or 10

12 3 and 8 and 11

13 (random$ or placebo$ or single blind$ or double blind$ or triple blind$).ti,ab.

14 RETRACTED ARTICLE/

15 13 or 14

16 (animal$ not human$).sh,hw.

17 (book or conference paper or editorial or letter or review).pt. not exp randomized controlled trial/

18 (random sampl$ or random digit$ or random effect$ or random survey or random regression).ti,ab. not exp randomized controlled trial/

19 15 not (16 or 17 or 18)

20 12 and 19

21 limit 20 to yr="2015 -Current" / for update: limit 20 to yr="2021 -Current"

mp=(multi-purpose field) title, abstract, heading word, drug trade name, original title, drug manufacturer, author keyword, floating subheading word, candidate term word

rn=CAS Registry Number (chemical names)
kw=Author keyword

BMJ Best Practice Study design search filters
Embase randomised controlled trial strategy
https://bestpractice.bmj.com/info/toolkit/learn-ebm/study-design-search-filters/

**Cochrane** CENTRAL
Cochrane Central Register of Controlled **Trials**

#1 (cholesterol* or lipid* or LDL):ti,ab,kw

#2 (statin or statins or (inhibit* NEAR/3 ("hmg coa" or "Hydroxymethylglutaryl CoA" or "Hydroxymethylglutaryl Coenzyme")) or atorvastatin or cerivastatin or crilvastatin or dalvastatin or fluindostatin or fluvastatin or glenvastatin or lovastatin or mevastatin or pitavastatin or pravastatin or rosuvastatin or simvastatin)

#3 (altoprev or altocor or baycol or canef or cranoc or compactin or crestor or lescol or lipitor or lipex or lipostat or livalo or locol or lochol or mevinolin or mevacor or mevalotin or mevinacor or monacolin or pravachol or pitava or pravachol or pravasin or zocor)

#4 (antichol* or antihyperchol* or hypochol* or hypolipidemic* or antihyperlipidemic* or anti-hyperlipidemic* or Ezetimibe or PCSK9 inhibitor* or Alirocumab or evolocumab or (non NEXT statin*))

#5 #2 or #3 or #4

#6 (((cardiovascular or heart or coronar* or cardiac) NEAR/3 (disease* or event* or attack* or mortalit* or death* or arrest*)) or cvd or cvds or CV-mortalit* or MACE or angina or ((heart or cardia* or myocard*) NEAR/3 (ischemi* or ischaemi* or fail* or insufficien*)) or ((myocard* or heart or cardiac) NEAR/3 (infarct* or attack*)) or (cerebrovascular* NEAR/3 (accident* or event*)) or cva or stroke* or ((brain or cerebral) NEAR/3 (ischemi* or ischaemi*)))

#7 #1 AND #5 AND #6

#8 Filter year range: 2015 to 2021 / for update: 2021 to 2023
