# Supplementary material for: Is there a shift from cardiovascular to cancer death in lipid-lowering trials? A systematic review and meta-analysis
Source: PLoS One. 2024 Feb 8;19(2):e0297852. doi: 10.1371/journal.pone.0297852 (PMC10852259; doi:10.1371/journal.pone.0297852)
Supplement: S1 File — (DOCX) [file pone.0297852.s004.docx]

**S1 Protocol.**

**Shift from cardiovascular death to cancer death in lipid-lowering trials– a systematic review and meta-analysis**

Citation

Alexandre Speierer, Sylvain Bétrisey, Martina Aeschbacher-Germann, Manuel Blum, Nicolas Rodondi, Elisavet Moutzouri, Cinzia Del Giovane. Shift from cardiovascular death to cancer death in lipid-lowering trials– a systematic review and meta-analysis. PROSPERO 2021 CRD42021271658 Available from: https://www.crd.york.ac.uk/prospero/display_record.php?ID=CRD42021271658

Review question

The purpose of this review is to assess a possible shift from CV death to cancer death in lipid-lowering trials among adults in primary and secondary prevention. Statin, ezetimibe and PSCK9 inhibitor will be included in this study as lipid-lowering therapy.

- AIM 1: to estimate the impact of lipid-lowering therapy use on cancer death in relation with the decrease of cardiovascular death

Searches

For the search, we will use all the eligible studies from the meta-analysis of the CTT Collaboration from 2019 (also the studies excluded for the final analysis due to missing IPD) and all trials mentioned on the CTT Collaboration homepage (04/2021) and perform an updated search. We will include also studies with other lipid-lowering treatments (ezetimibe and PSCK9 inhibitors), as the effect could be LDL-related.

- Following electronic resources will be searched: Cochrane Library, EMBASE, MEDLINE, PubMed

- The search strategy will be developed in cooperation with trained librarians.

- Publication period: in the last 6 years (01.2015-30.04.2021).

- We decided on conducting the search only for the last 6 years (01.2015-04.2021) since the CTT Collaboration published their last meta-analysis on large statin trials in 2019. In this meta-analysis the latest included trial was published in 2016. And for non-statin trials the first published trial on cardiovascular outcomes was published in 2015. And for Ezetimibe the landmark trial on cardiovascular outcomes is IMPROVE-IT , published in 2015.

- All studies in English

- unpublished studies: not included

Types of study to be included

Inclusion criteria: randomized controlled trials (RCTs) including >1000 participants on lipid-lowering medication use as defined above with any cardiovascular clinical events as primary endpoints in primary and secondary prevention. In English.

Exclusion criteria: design other than RCT.

Condition or domain being studied

Adult patients randomized to lipid-lowering medications, defined as trials of statins, ezetimibe and PCSK-9 inhibitor -because these drugs have been shown to reduce cardiovascular risk- in primary or secondary prevention with cardiovascular disease and/or mortality as primary outcomes.

Participants/population

Inclusion

- Adults patients

- Statin, ezetimibe or PSCK9 inhibitor intake for primary and secondary prevention of cardiovascular disease and mortality

Exclusion

- Adults taking statin, ezetimibe or PSCK9 inhibitor for another reason than primary and secondary prevention of cardiovascular disease

Intervention(s), exposure(s)

Any statin treatment, ezetimibe or PSCK9 inhibitor for primary and secondary prevention of cardiovascular disease

Comparator(s)/control

Placebo, active control, no treatment

Main outcome(s)

Primary outcomes:

- Cardiovascular death

- Cancer death

Measures of effect

- Hazard Ratio

- Risk Ratio

Additional outcome(s)

Study characteristics (such as lead author, publication year, sample size, setting, intervention and comparator, duration of follow-up, funding source), participant characteristics (age, sex, multimorbidity) and outcome data.

We will extract number of cardiovascular deaths, cancer death in the intervention and control group. If some data are not available from included studies, reported data in CCT may be used.

Data extraction (selection and coding)

The PRISMA statement will be followed at all stages.

Duplicates will be removed using reference management software. By using the software Rayaan, two authors will first screen the study’s title and abstract for eligibility. In a second step the same two authors will screen the full-text of studies eligible from step one. The two first step will be done independently. Disagreement will be discussed and where no consensus is found a third independent person will be consulted.

In the case of multiple publications of a study, we will take the one with the most participants we are interested in. We will exclude duplicate data, secondary subgroup trial data analyses, posttrial follow up studies and studies with LDL as primary endpoint.

We will list excluded full-text studies and give the reason for their exclusion.

Risk of bias (quality) assessment

We will use RoB 2.0 according to Cochrane Collaboration 2019

Strategy for data synthesis

We will report absolute risk difference (ARD) estimates, calculated using as baseline the proportion of patients with an event in the control arm (i.e., placebo/no treatment) of the included studies, and applying the RR estimated in the metaanalysis to compute the absolute difference between the intervention and control arms within GRADEPro. With this analysis we aimed to assess the shift from cardiovascular death to cancer death.

As second, meta-analysis for aggregated survival data with competing risks. Cancer death will be the outcome of interest and cardiovascular death the competing event. We will estimate the CIF (cumulative incidence function) and the CIF ratio to assess the treatment effect and pool CIF ratios from included studies. We will calculate HR for cancer death considering the cardiovascular death as competing risk. We will also calculate the risk ratio for cancer death and cardiovascular death from each study. We will present the results of the 2 outcomes in a scatter plot to allow us a simple graphical visualization of the potential shift or correlation.

We will report the results as HR and RR accompanied by their 95% confidence intervals. We will assess heterogeneity by visual inspection of the forest plots and by estimating I². We will also assess the publication bias with funnel plots. All analyses will be conducted in STATA.

Analysis of subgroups or subsets

Subgroup analysis will be used to explore possible sources of heterogeneity: preplanned variables to explore are age, sex, primary versus secondary prevention, lipid lowering treatment (statins, ezetimibe, PCSK9 inhibitors)

Contact details for further information

Elisavet Moutzouri

elisavet.moutzouri@extern.insel.ch

Organisational affiliation of the review

Institute of Primary Health Care (BIHAM), University of Bern, Bern, Switzerland. Mittelstrasse 43, 3012 Bern, Switzerland

<http://www.biham.unibe.ch>

Review team members and their organisational affiliations

Mr Alexandre Speierer. Institute of Primary Health Care (BIHAM)

Mr Sylvain Bétrisey. Institute of Primary Health Care (BIHAM)

Mrs Martina Aeschbacher-Germann. Institute of Primary Health Care (BIHAM)

Dr Manuel Blum. Institute of Primary Health Care (BIHAM)

Professor Nicolas Rodondi. Institute of Primary Health Care (BIHAM)

Dr Elisavet Moutzouri. Institute of Primary Health Care (BIHAM)

Dr Cinzia Del Giovane. Institute of Primary Health Care (BIHAM)

Collaborators

Dr Baris Gencer. Cardiology Division, Geneva University Hospitals, University of Geneva

Type and method of review

Systematic review

Anticipated or actual start date

01 March 2021

Anticipated completion date

30 November 2021

Funding sources/sponsors

None

Grant number(s)

State the funder, grant or award number and the date of award

None

Conflicts of interest

Language

English

Country

Switzerland

Stage of review

Review Ongoing

Subject index terms status

Subject indexing assigned by CRD

Subject index terms

Cardiovascular Diseases; Humans; Hydroxymethylglutaryl-CoA Reductase Inhibitors; Lipids; Neoplasms

Date of registration in PROSPERO

04 September 2021

Date of first submission

04 August 2021

Stage of review at time of this submission

**Stage:** **Started** **Completed**

Preliminary searches Yes Yes

Piloting of the study selection process Yes Yes

Formal screening of search results against eligibility criteria Yes No

Data extraction No No

Risk of bias (quality) assessment Yes No

Data analysis No No

*The record owner confirms that the information they have supplied for this submission is accurate and complete and they understand that deliberate provision of inaccurate information or omission of data may be construed as scientific misconduct.*

*The record owner confirms that they will update the status of the review when it is completed and will add publication details in due course.*

Versions

04 September 2021

04 September 2021
