# Supplementary figures and images for: Is there a shift from cardiovascular to cancer death in lipid-lowering trials? A systematic review and meta-analysis
Source: PLoS One. 2024 Feb 8;19(2):e0297852. doi: 10.1371/journal.pone.0297852 (PMC10852259; doi:10.1371/journal.pone.0297852)

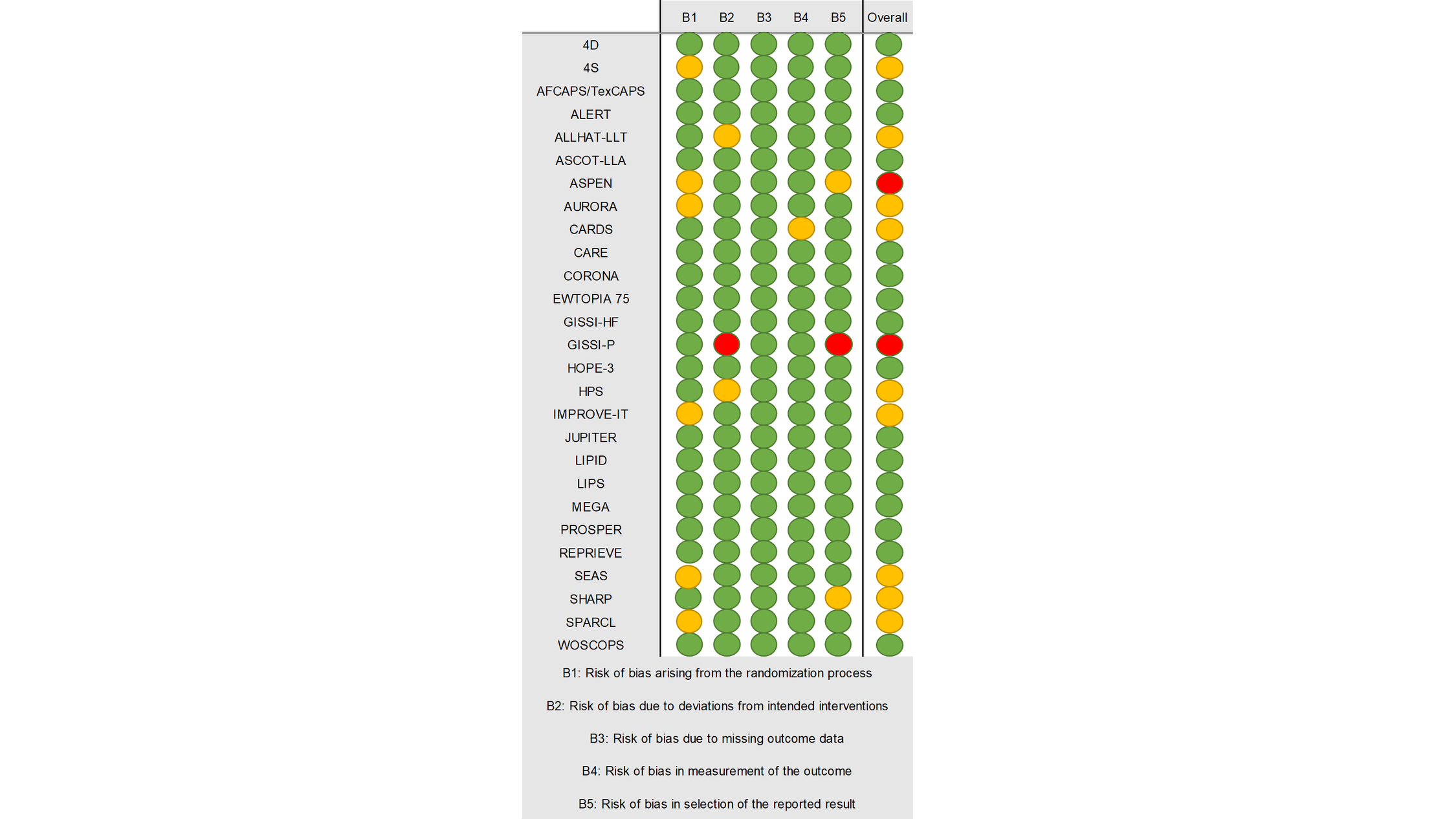

Supplement: S1 Fig — 1 Green represents a low, orange an intermediate, and red a high risk of bias. (TIF) [file pone.0297852.s005.tif]

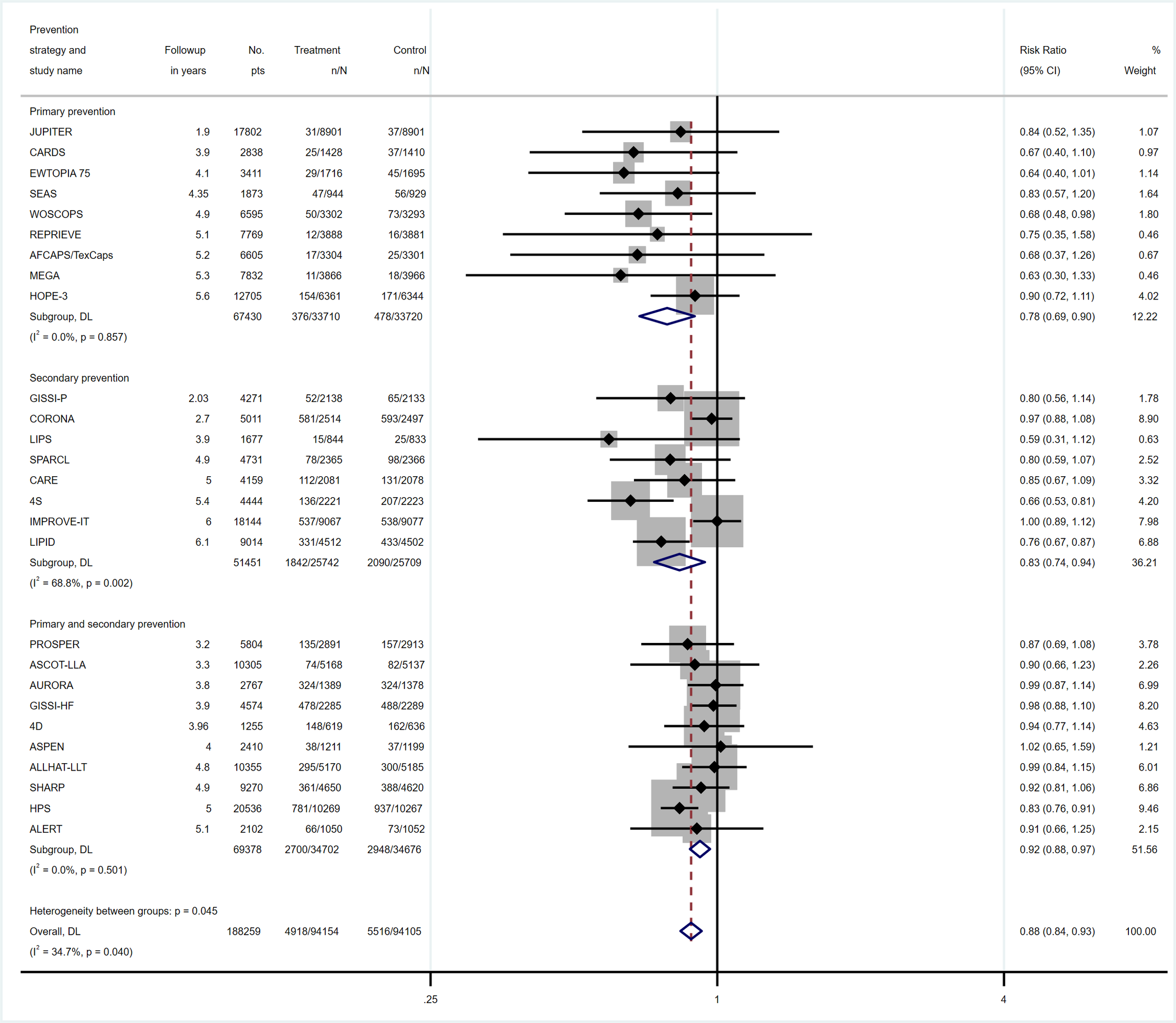

Supplement: S2 Fig — 1 pts = participants, CI = confidence interval, 1 Weights and between-subgroup heterogeneity test are from random-effects model. (TIF) [file pone.0297852.s006.tif]

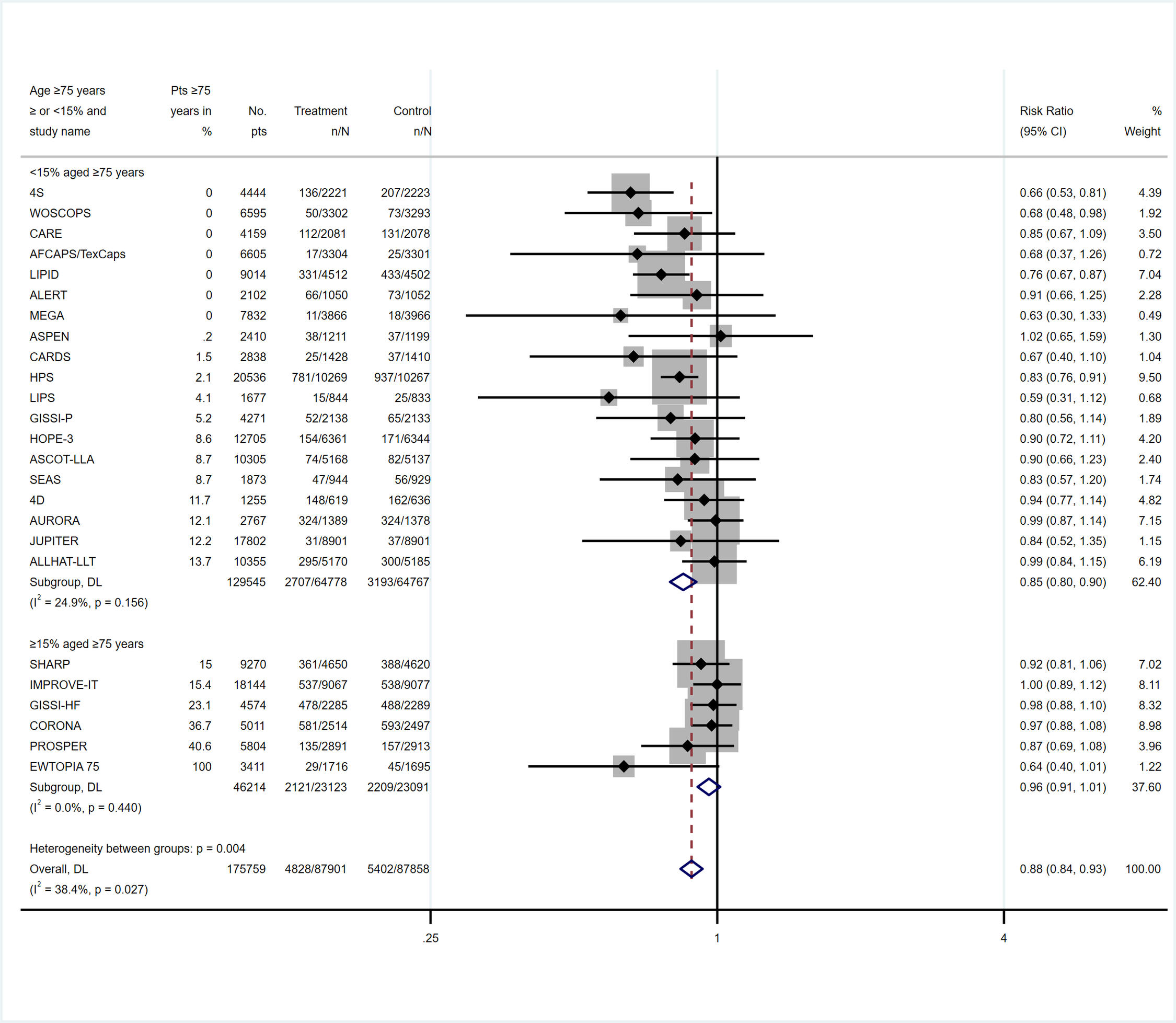

Supplement: S3 Fig — 1, 2 pts = participants, CI = confidence interval, 1Weights and between-subgroup heterogeneity test are from random-effects model, 2The SPARCL and REPRIEVE trial did not report the prevalence of participants aged ≥75 years. (TIF) [file pone.0297852.s007.tif]

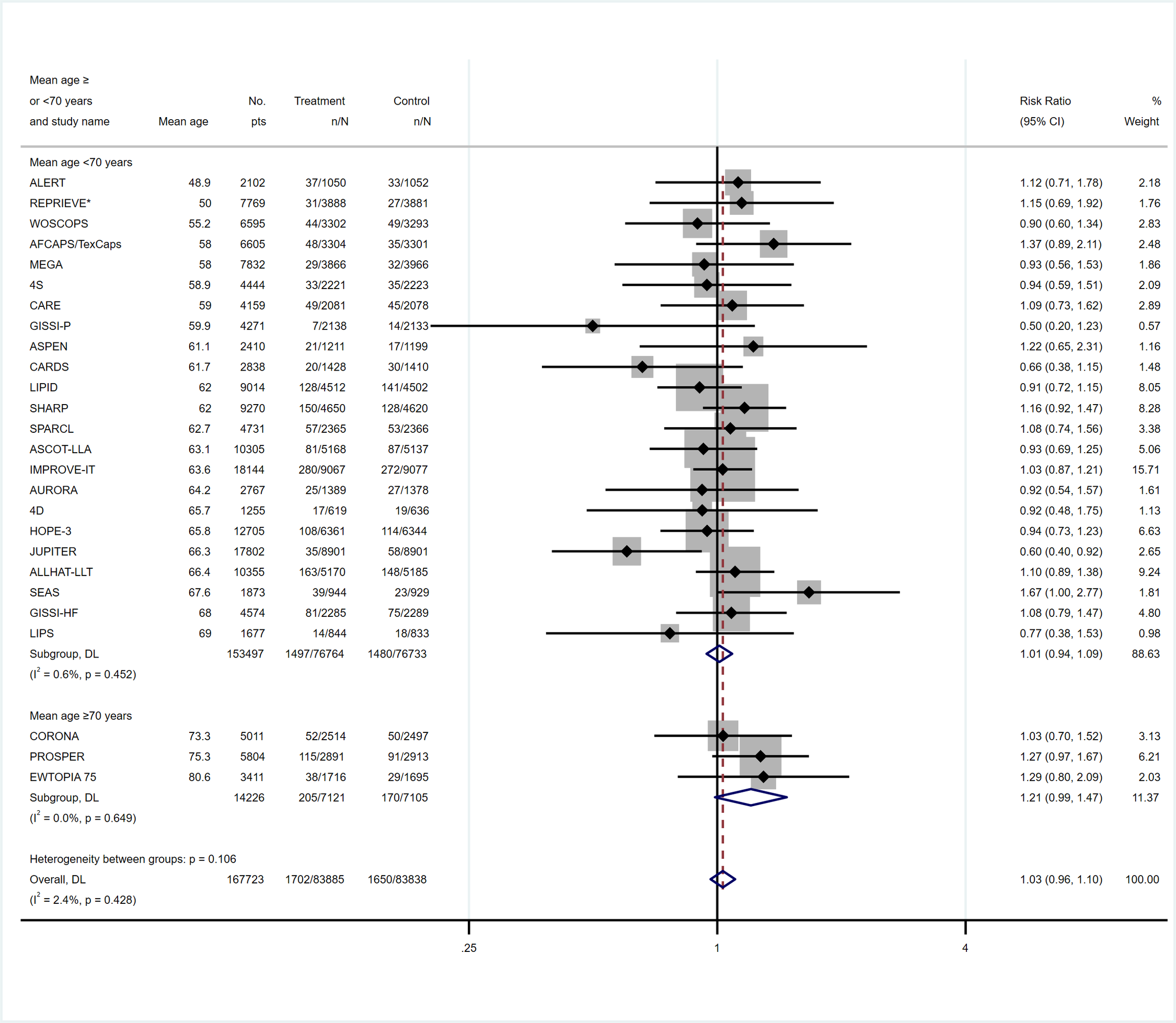

Supplement: S4 Fig — pts = participants, CI = confidence interval, 1 Weights and between-subgroup heterogeneity test are from random-effects model, 2The HPS trial did not report the mean age. (TIF) [file pone.0297852.s008.tif]

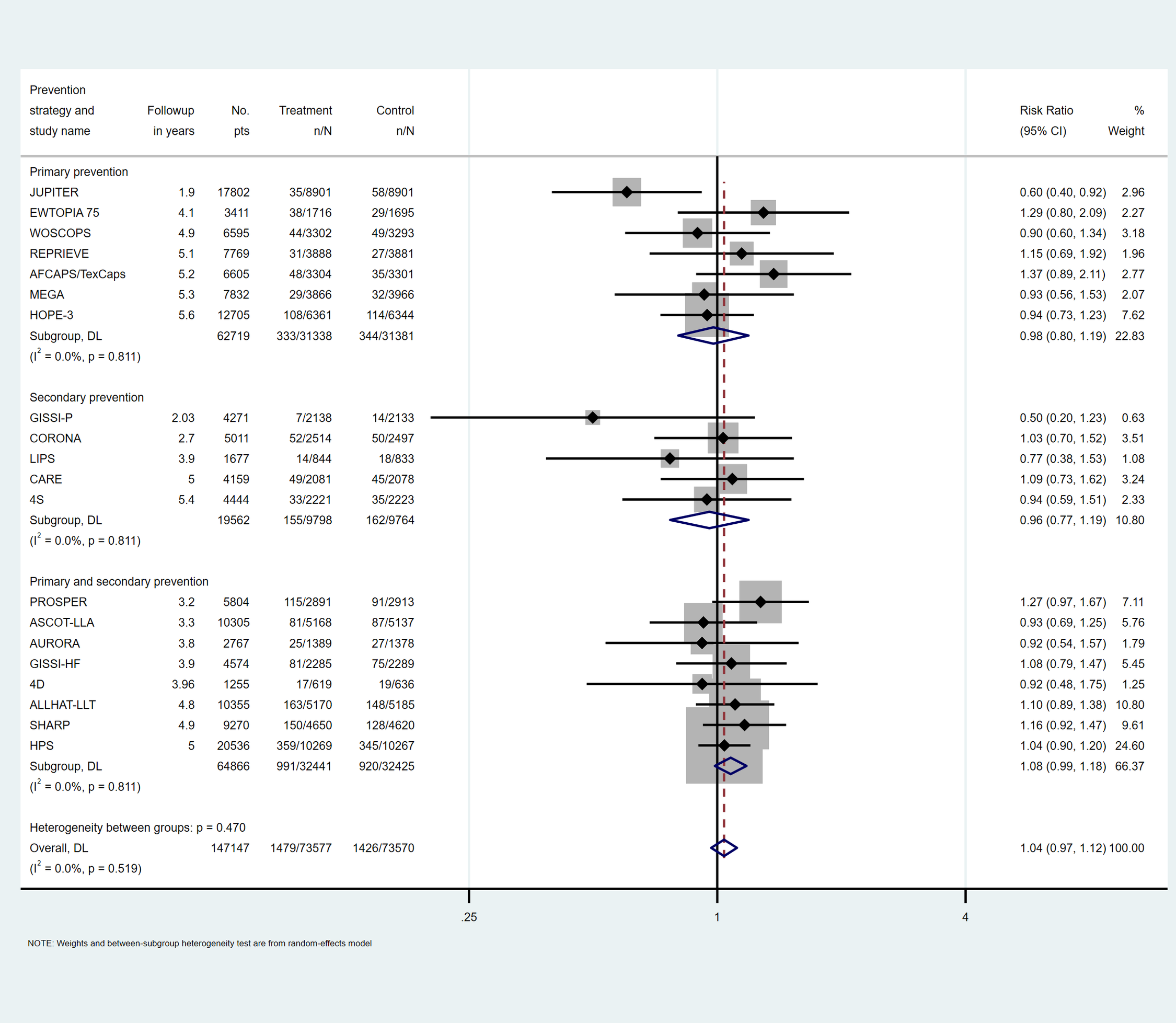

Supplement: S5 Fig — pts = participants, CI = confidence interval, 1 Weights and between-subgroup heterogeneity test are from random-effects model. (TIF) [file pone.0297852.s009.tif]

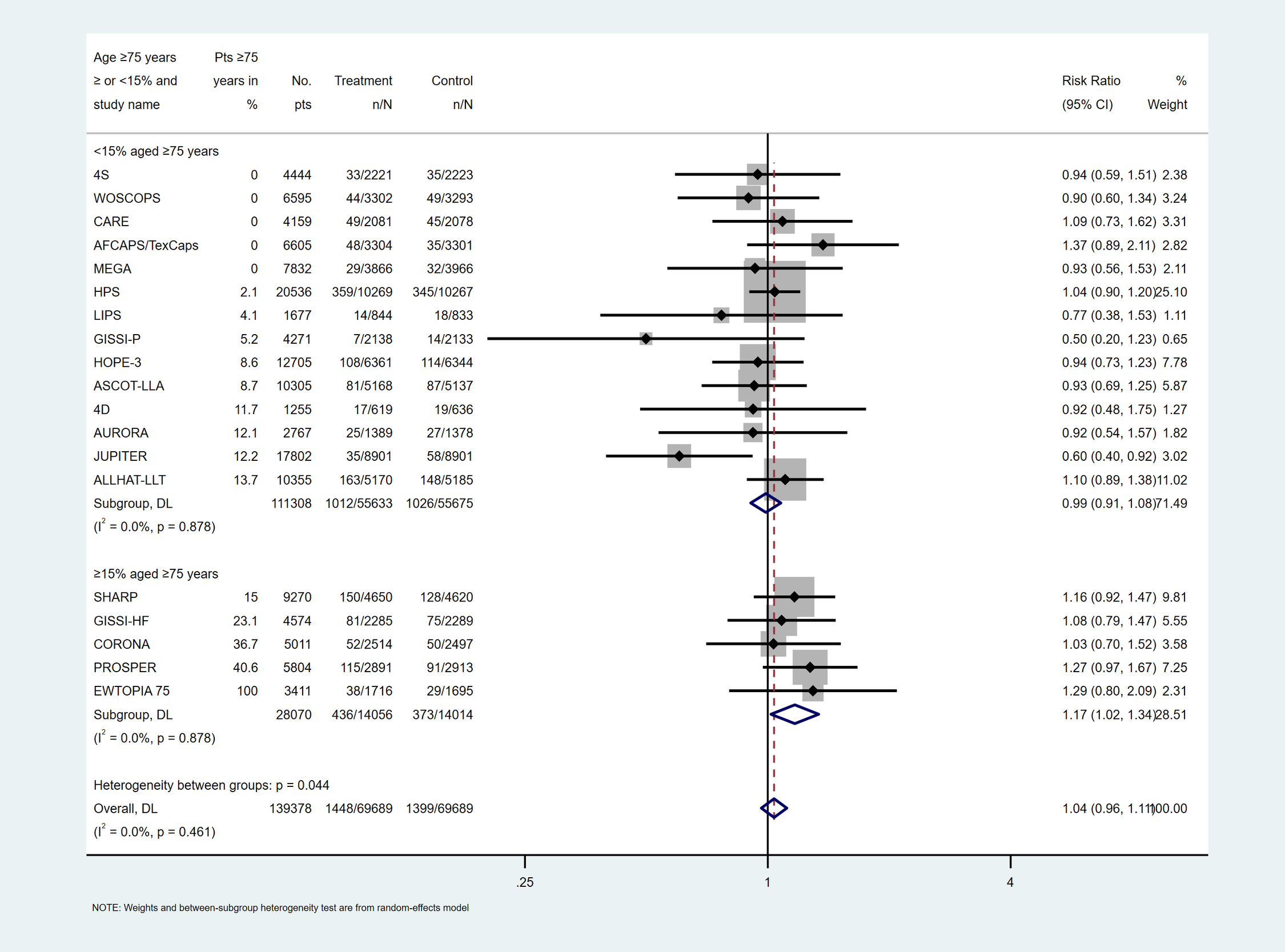

Supplement: S6 Fig — pts = participants, CI = confidence interval, 1The REPRIEVE trial did not report the prevalence of participants aged ≥75 years. (TIF) [file pone.0297852.s010.tif]

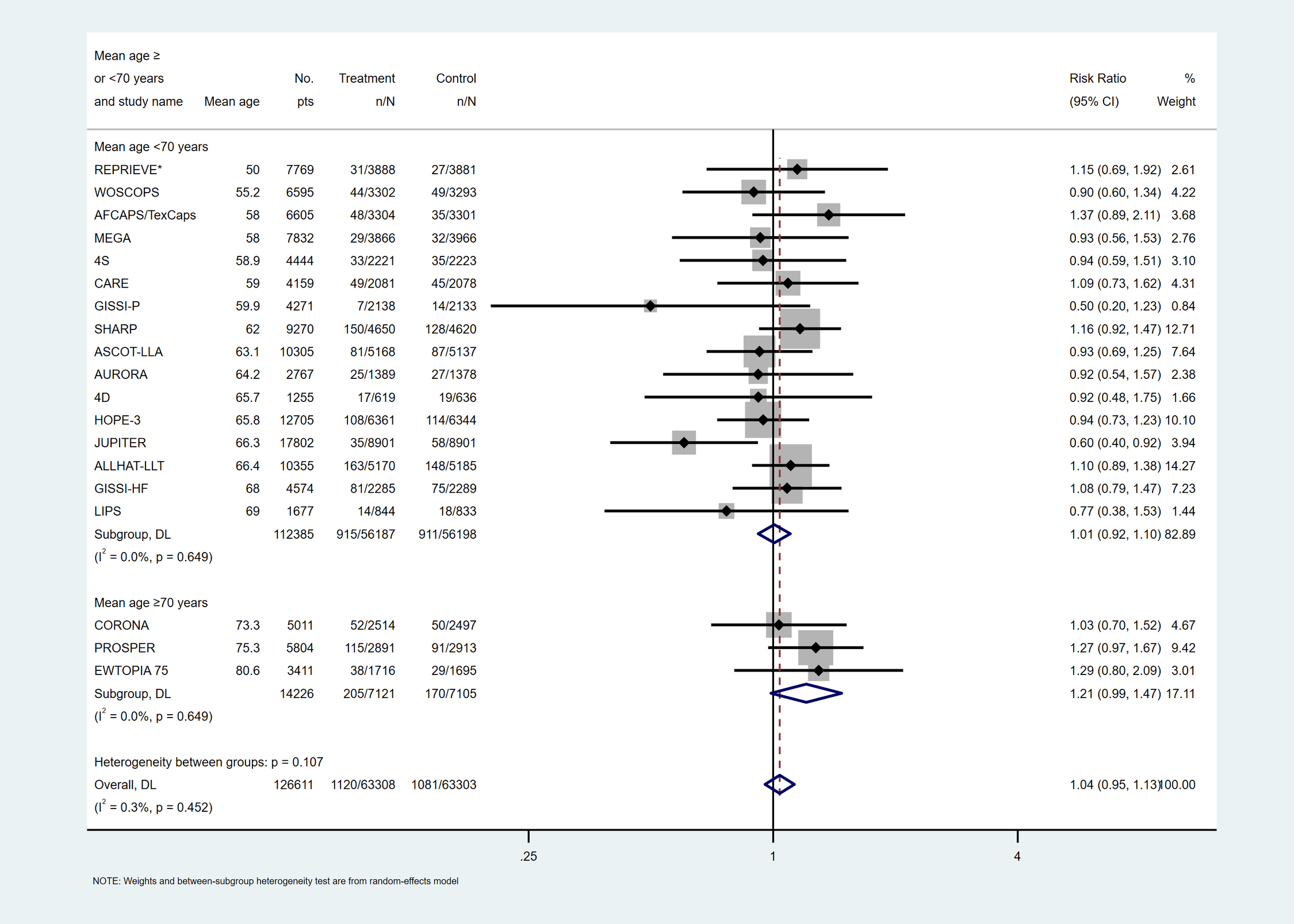

Supplement: S7 Fig — pts = participants, CI = confidence interval, 1Weights and between-subgroup heterogeneity test are from random-effects model, 2The HPS trial did not report the mean age. (TIF) [file pone.0297852.s011.tif]
