# Supplementary material for: Is there a shift from cardiovascular to cancer death in lipid-lowering trials? A systematic review and meta-analysis
Source: PLoS One. 2024 Feb 8;19(2):e0297852. doi: 10.1371/journal.pone.0297852 (PMC10852259; doi:10.1371/journal.pone.0297852)
Supplement: S1 Table — LLT = Lipid-lowering treatment, CV = cardiovascular. (DOCX) [file pone.0297852.s012.docx]

| **S1 Table**. | | | | | | | | | | | | | | |
| --- | --- | --- | --- | --- | --- | --- | --- | --- | --- | --- | --- | --- | --- | --- |
| **Control arm: Placebo, no treatment or usual care** | | | | | | | | | | | | | | |
| **Trial Name** | **Primary or Secondary Prevention** | **Intervention** | **Mean age (years)** | **Men (%)** | **Follow-up (years)** | **Overall (N)** |  | **LLT Group** | | |  | **Control Group** | | |
|  |  |  |  |  |  |  |  | **Cancer death (N)** | **CV death (N)** | **Overall (N)** |  | **Cancer death (N)** | **CV death (N)** | **Overall (N)** |
| 4S (1) | Secondary | 20mg Simvastatin | 58.9 | 72 | 5.4 | 4444 |  | 33 | 136 | 2221 |  | 35 | 207 | 2223 |
| WOSCOPS (2) | Primary | 40mg Pravastatin | 55.2 | 100 | 4.9 | 6595 |  | 44 | 50 | 3302 |  | 49 | 73 | 3293 |
| CARE (3) | Secondary | 40mg Pravastatin | 59 | 86 | 5 | 4159 |  | 49 | 112 | 2081 |  | 45 | 131 | 2078 |
| AFCAPS/TexCaps (4) | Primary | 20-40mg Lovastatin | 58 | 85 | 5.2 | 6605 |  | 48 | 17 | 3304 |  | 35 | 25 | 3301 |
| LIPID (5) | Secondary | 40mg Pravastatin | 62 | 83 | 6.1 | 9014 |  | 128 | 331 | 4512 |  | 141 | 433 | 4502 |
| GISSI-P (6) | Secondary | 20mg Pravastatin | 59.9 | 86.3 | 2.03 | 4271 |  | 7 | 52 | 2138 |  | 14 | 65 | 2133 |
| HPS (7) | Primary & Secondary | 40mg Simvastatin | NR | 75.3 | 5 | 20536 |  | 359 | 781 | 10269 |  | 345 | 937 | 10267 |
| LIPS (8) | Secondary | 80mg Fluvastatin | 69 | 83.8 | 3.9 | 1677 |  | 14 | 15 | 844 |  | 18 | 25 | 833 |
| PROSPER (9) | Primary & Secondary | 40mg Pravastatin | 75.3 | 48.3 | 3.2 | 5804 |  | 115 | 135 | 2891 |  | 91 | 157 | 2913 |
| ALERT (10) | Primary & Secondary | 40mg Fluvastatin | 48.9 | 66 | 5.1 | 2102 |  | 37 | 66 | 1050 |  | 33 | 73 | 1052 |
| ASCOT-LLA (11) | Primary & Secondary | 10mg Atorvastatin | 63.1 | 91.2 | 3.3 | 10305 |  | 81 | 74 | 5168 |  | 87 | 82 | 5137 |
| CARDS (12) | Primary | 10mg Atorvastatin | 61.7 | 68 | 3.9 | 2838 |  | 20 | 25 | 1428 |  | 30 | 37 | 1410 |
| 4D (13) | Primary & Secondary | 20mg Atorvastatin | 65.7 | 54 | 3.96 | 1255 |  | 17 | 148 | 619 |  | 19 | 162 | 636 |
| ASPEN (14) | Primary & Secondary | 10mg Atorvastatin | 61.1 | 66.3 | 4 | 2410 |  | 21 | 38 | 1211 |  | 17 | 37 | 1199 |
| SPARCL (15) | Secondary | 80mg Atorvastatin | 62.7 | 59.7 | 4.9 | 4731 |  | 57 | 78 | 2365 |  | 53 | 98 | 2366 |
| MEGA (16) | Primary | 10-20mg Pravastatin (TCL>5.69mmol/l) | 58 | 31.6 | 5.3 | 7832 |  | 29 | 11 | 3866 |  | 32 | 18 | 3966 |
| CORONA (17) | Secondary | 10mg Rosuvastatin | 73.3 | 76 | 2.7 | 5011 |  | 52 | 581 | 2514 |  | 50 | 593 | 2497 |
| GISSI-HF (18) | Primary & Secondary | 10mg Rosuvastatin | 68 | 77.4 | 3.9 | 4574 |  | 81 | 478 | 2285 |  | 75 | 488 | 2289 |
| JUPITER (19) | Primary | 20mg Rosuvastatin | 66.3 | 61.8 | 1.9 | 17802 |  | 35 | 31 | 8901 |  | 58 | 37 | 8901 |
| AURORA (20) | Primary & Secondary | 10mg Rosuvastatin | 64.2 | 62.9 | 3.8 | 2776 |  | 25 | 324 | 1389 |  | 27 | 324 | 1378 |
| HOPE-3 (21) | Primary | 10mg Rosuvastatin | 65.8 | 53.8 | 5.6 | 12705 |  | 108 | 154 | 6361 |  | 114 | 171 | 6344 |
| ALLHAT-LLT (22) | Primary & Secondary | 40mg Pravastatin | 66.4 | 51 | 4.8 | 10355 |  | 163 | 295 | 5170 |  | 148 | 300 | 5185 |
| REPRIEVE (23) | Primary | 4mg Pitavastatin | 50 (median) | 69 | 5.1 | 7769 |  | 31 | 12 | 3888 |  | 27 | 16 | 3881 |
| SEAS (24) | Primary | 40mg Simvastatin + 10mg Ezetimibe | 67.6 | 61.4 | 4.35 | 1873 |  | 39 | 47 | 944 |  | 23 | 56 | 929 |
| SHARP (25) | Primary & Secondary | 20mg Simvastatin + 10mg Ezetimibe | 62 | 62.6 | 4.9 | 9270 |  | 150 | 361 | 4650 |  | 128 | 388 | 4620 |
| IMPROVE-IT (26) | Secondary | 40mg Simvastatin +10mg Ezetimibe | 63.6 | 75.7 | 6 | 18144 |  | 280 | 537 | 9067 |  | 272 | 538 | 9077 |
| EWTOPIA 75 (27) | Primary | 10mg Ezetimibe | 80.6 | 25.5 | 4.1 | 3411 |  | 38 | 29 | 1716 |  | 29 | 45 | 1695 |

1. Randomised trial of cholesterol lowering in 4444 patients with coronary heart disease: the Scandinavian Simvastatin Survival Study (4S). Lancet. 1994;344(8934):1383-9.

2. Shepherd J, Cobbe SM, Ford I, Isles CG, Lorimer AR, Macfarlane PW, et al. Prevention of Coronary Heart Disease with Pravastatin in Men with Hypercholesterolemia. New England Journal of Medicine. 1995;333(20):1301-8.

3. Sacks FM, Pfeffer MA, Moye LA, Rouleau JL, Rutherford JD, Cole TG, et al. The Effect of Pravastatin on Coronary Events after Myocardial Infarction in Patients with Average Cholesterol Levels. New England Journal of Medicine. 1996;335(14):1001-9.

4. Downs JR, Clearfield M, Weis S, Whitney E, Shapiro DR, Beere PA, et al. Primary Prevention of Acute Coronary Events With Lovastatin in Men and Women With Average Cholesterol LevelsResults of AFCAPS/TexCAPS. JAMA. 1998;279(20):1615-22.

5. Prevention of cardiovascular events and death with pravastatin in patients with coronary heart disease and a broad range of initial cholesterol levels. N Engl J Med. 1998;339(19):1349-57.

6. Investigators G-P. Results of the low-dose (20 mg) pravastatin GISSI Prevenzione trial in 4271 patients with recent myocardial infarction: do stopped trials contribute to overall knowledge? GISSI Prevenzione Investigators (Gruppo Italiano per lo Studio della Sopravvivenza nell'Infarto Miocardico). Ital Heart J. 2000;1:810-20.

7. MRC/BHF Heart Protection Study of cholesterol lowering with simvastatin in 20 536 high-risk individuals: a randomised placebocontrolled trial. The Lancet. 2002;360(9326):7-22.

8. Serruys PWJC, de Feyter P, Macaya C, Kokott N, Puel J, Vrolix M, et al. Fluvastatin for Prevention of Cardiac Events Following Successful First Percutaneous Coronary InterventionA Randomized Controlled Trial. JAMA. 2002;287(24):3215-22.

9. Shepherd J, Blauw GJ, Murphy MB, Bollen EL, Buckley BM, Cobbe SM, et al. Pravastatin in elderly individuals at risk of vascular disease (PROSPER): a randomised controlled trial. The Lancet. 2002;360(9346):1623-30.

10. Holdaas H, Fellström B, Jardine AG, Holme I, Nyberg G, Fauchald P, et al. Effect of fluvastatin on cardiac outcomes in renal transplant recipients: a multicentre, randomised, placebo-controlled trial. Lancet. 2003;361(9374):2024-31.

11. Sever PS, Dahlöf B, Poulter NR, Wedel H, Beevers G, Caulfield M, et al. Prevention of coronary and stroke events with atorvastatin in hypertensive patients who have average or lower-than-average cholesterol concentrations, in the Anglo-Scandinavian Cardiac Outcomes Trial--Lipid Lowering Arm (ASCOT-LLA): a multicentre randomised controlled trial. Lancet. 2003;361(9364):1149-58.

12. Colhoun HM, Betteridge DJ, Durrington PN, Hitman GA, Neil HA, Livingstone SJ, et al. Primary prevention of cardiovascular disease with atorvastatin in type 2 diabetes in the Collaborative Atorvastatin Diabetes Study (CARDS): multicentre randomised placebo-controlled trial. Lancet. 2004;364(9435):685-96.

13. Wanner C, Krane V, März W, Olschewski M, Mann JFE, Ruf G, et al. Atorvastatin in Patients with Type 2 Diabetes Mellitus Undergoing Hemodialysis. New England Journal of Medicine. 2005;353(3):238-48.

14. Knopp RH, d'Emden M, Smilde JG, Pocock SJ. Efficacy and safety of atorvastatin in the prevention of cardiovascular end points in subjects with type 2 diabetes: the Atorvastatin Study for Prevention of Coronary Heart Disease Endpoints in non-insulin-dependent diabetes mellitus (ASPEN). Diabetes Care. 2006;29(7):1478-85.

15. Amarenco P, Bogousslavsky J, Callahan A, 3rd, Goldstein LB, Hennerici M, Rudolph AE, et al. High-dose atorvastatin after stroke or transient ischemic attack. New England Journal of Medicine. 2006;355(6):549-59.

16. Nakamura H, Arakawa K, Itakura H, Kitabatake A, Goto Y, Toyota T, et al. Primary prevention of cardiovascular disease with pravastatin in Japan (MEGA Study): a prospective randomised controlled trial. Lancet. 2006;368(9542):1155-63.

17. Kjekshus J, Apetrei E, Barrios V, Böhm M, Cleland JGF, Cornel JH, et al. Rosuvastatin in Older Patients with Systolic Heart Failure. New England Journal of Medicine. 2007;357(22):2248-61.

18. Tavazzi L, Maggioni AP, Marchioli R, Barlera S, Franzosi MG, Latini R, et al. Effect of rosuvastatin in patients with chronic heart failure (the GISSI-HF trial): a randomised, double-blind, placebo-controlled trial. Lancet. 2008;372(9645):1231-9.

19. Ridker PM, Danielson E, Fonseca FAH, Genest J, Gotto AM, Kastelein JJP, et al. Rosuvastatin to Prevent Vascular Events in Men and Women with Elevated C-Reactive Protein. New England Journal of Medicine. 2008;359(21):2195-207.

20. Fellström BC, Jardine AG, Schmieder RE, Holdaas H, Bannister K, Beutler J, et al. Rosuvastatin and Cardiovascular Events in Patients Undergoing Hemodialysis. New England Journal of Medicine. 2009;360(14):1395-407.

21. Yusuf S, Bosch J, Dagenais G, Zhu J, Xavier D, Liu L, et al. Cholesterol Lowering in Intermediate-Risk Persons without Cardiovascular Disease. New England Journal of Medicine. 2016;374(21):2021-31.

22. Han BH, Sutin D, Williamson JD, Davis BR, Piller LB, Pervin H, et al. Effect of Statin Treatment vs Usual Care on Primary Cardiovascular Prevention Among Older Adults: The ALLHAT-LLT Randomized Clinical Trial. JAMA Intern Med. 2017;177(7):955-65.

23. Grinspoon SK, Fitch KV, Zanni MV, Fichtenbaum CJ, Umbleja T, Aberg JA, et al. Pitavastatin to Prevent Cardiovascular Disease in HIV Infection. New England Journal of Medicine. 2023;389(8):687-99.

24. Rossebø AB, Pedersen TR, Boman K, Brudi P, Chambers JB, Egstrup K, et al. Intensive Lipid Lowering with Simvastatin and Ezetimibe in Aortic Stenosis. New England Journal of Medicine. 2008;359(13):1343-56.

25. Baigent C, Landray MJ, Reith C, Emberson J, Wheeler DC, Tomson C, et al. The effects of lowering LDL cholesterol with simvastatin plus ezetimibe in patients with chronic kidney disease (Study of Heart and Renal Protection): a randomised placebo-controlled trial. Lancet. 2011;377(9784):2181-92.

26. Cannon CP, Blazing MA, Giugliano RP, McCagg A, White JA, Theroux P, et al. Ezetimibe Added to Statin Therapy after Acute Coronary Syndromes. New England Journal of Medicine. 2015;372(25):2387-97.

27. Ouchi Y, Sasaki J, Arai H, Yokote K, Harada K, Katayama Y, et al. Ezetimibe Lipid-Lowering Trial on Prevention of Atherosclerotic Cardiovascular Disease in 75 or Older (EWTOPIA 75): A Randomized, Controlled Trial. Circulation. 2019;140(12):992-1003.
