# Supplementary material for: Is there a shift from cardiovascular to cancer death in lipid-lowering trials? A systematic review and meta-analysis
Source: PLoS One. 2024 Feb 8;19(2):e0297852. doi: 10.1371/journal.pone.0297852 (PMC10852259; doi:10.1371/journal.pone.0297852)
Supplement: S2 Table — RR = relative risk, CI = confidence interval. (DOCX) [file pone.0297852.s013.docx]

|  | **Trials with both primary and secondary prevention** | | | **All trials** | | |
| --- | --- | --- | --- | --- | --- | --- |
| **Exclusion/Inclusion** | **N trials** | **RR (95% CI)** | **I^2^ in %** | **N trials** | **RR (95% CI)** | **I^2^ in %** |
| **No exclusion criteria applied** | 10 | 1.08 (0.99 – 1.18) | 0 | 27 | 1.03 (0.97 – 1.10) | 0 |
| **High risk of bias excluded** | 9 | 1.08 (0.99 – 1.18) | 0 | 25 | 1.04 (0.97 – 1.10) | 0 |
| **Premature end excluded** | 10 | 1.08 (0.99 – 1.18) | 0 | 20 | 1.05 ( 0.98 – 1.12) | 0 |
| **Only statins included** | 9 | 1.07 (0.97 – 1.17) | 0 | 23 | 1.01 (0.94 – 1.08) | 0 |

**S2 Table.**
